# Hypoglycemic and Antidiabetic Effect of *Pleurotus sajor-caju* Aqueous Extract in Normal and Streptozotocin-Induced Diabetic Rats

**DOI:** 10.1155/2015/214918

**Published:** 2015-11-23

**Authors:** Sze Han Ng, Mohd Shazwan Mohd Zain, Fatariah Zakaria, Wan Rosli Wan Ishak, Wan Amir Nizam Wan Ahmad

**Affiliations:** ^1^Nutrition Program, School of Health Sciences, Universiti Sains Malaysia, Health Campus, 16150 Kubang Kerian, Kelantan, Malaysia; ^2^Biomedicine Program, School of Health Sciences, Universiti Sains Malaysia, Health Campus, 16150 Kubang Kerian, Kelantan, Malaysia

## Abstract

*Introduction*.* Pleurotus sajor-caju* (PSC) is an edible oyster mushroom featuring high nutritional values and pharmacological properties.* Objective*. To investigate the hypoglycemic and antidiabetic effects of single and repeated oral administration of PSC aqueous extract in normal and diabetic rats.* Materials and Methods*. A single dose of 500, 750, or 1000 mg/kg of the PSC extract was given to experimental rats to determine the effects on blood glucose (BG) and oral glucose tolerance test (OGTT). The effective dose (750 mg/kg) of PSC extract was repeatedly administrated daily for 21 days in diabetic rats to examine its antidiabetic effects in terms of BG control, body weight, urine sugar, HbA1c, and several serum profiles.* Results*. The dose of 750 mg/kg showed the most significant BG reduction (23.5%) in normal rats 6 hours after administration in BG study (*p* < 0.05). In OGTT study, the same dose produced a maximum BG fall of 41.3% in normal rats and 36.5% in diabetic rats 3 hours after glucose administration. In 21-day study, treated diabetic rats showed significant improvement in terms of fasting BG, body weight, and urine sugar as compared to control diabetic rats.* Conclusion*. The study evidenced scientifically the beneficial use of PSC as an alternative medicine in diabetes management.

## 1. Introduction

Diabetes mellitus (DM) is a debilitating chronic metabolic disorder featured by hyperglycemia due to defects in insulin secretion, insulin action, or both [1]. Presently, there is a rapid rise in the prevalence of DM, with the number of diabetic individuals estimated to doubly increase from 171 million in 2000 to 366 million in 2030 [2]. It is the major cause of morbidity and mortality worldwide as it may lead to health complications and affect quality of life [3].

Management of diabetes basically initiates with a modification in diet and exercise. Nonetheless, most diabetic patients eventually require pharmacotherapy, such as injection of insulin and/or administration of oral antidiabetic drugs. Many oral hypoglycemic drugs such as biguanides, *α*-glucosidase inhibitors, sulphonylurea, and thiazolidinediones are available for the DM treatment. These synthetic drugs are the mainstay of treatment of diabetes and are effective in controlling hyperglycemia but they are not free from harmful side effects such as hypoglycemia, hepatic toxicity, weight increase, abdomen enlargement, and gastrointestinal discomfort [4, 5]. Therefore, development of safe and effective oral hypoglycemic agents from medicinal plants to manage DM without side effects is of great interest recently and is strongly recommended by WHO [6]. In recent years, traditional Chinese medicine such as* Ginkgo biloba* extract and crude extract from natural products such as Leguminosae, Cucurbitaceae, and Araliaceae have been established scientifically to have antidiabetic effects [7, 8].

*Pleurotus sajor-caju* (PSC), one of the species from* Pleurotus *spp., is an edible oyster mushroom firstly discovered in India and characterized by a white spore print and gills attachment as well as eccentric stip occasionally [9]. It grows on trunks and stumps of deciduous trees in tropical and subtropical rainforests and could be artificially cultivated on various agricultural residues [10]. It ranks second most popular cultivated mushrooms after button mushroom and accounted for 14.2% of the total world mushroom production [11]. For centuries, it has been widely used for culinary and medicinal purpose due to its pleasant taste and pharmacological properties.

This prominent edible oyster mushroom is claimed to possess considerable importance in the human diet as it shows favourable dietetic properties. It is attributed to its high protein, minerals (calcium, phosphorus, and iron), B vitamins (thiamin, riboflavin, and folic acid), and dietary fiber content [12]. On the other hand, it is low in fat content and calorific value (kcal/g) and completely devoid of starch; hence it could be an excellent inclusion in the diet of individuals with hyperlipidemia and diabetes [13]. Therefore, it is considered to be advantageous in the prevention and management of DM. Previously, attention has been focused primarily on its immune modulating, hypotensive, hypocholesterolemic, and antitumor properties [14, 15]. Nevertheless, lack of scientific information is available on its antidiabetic properties.

Thus, it was worthwhile to find out the scientific basis for its utilization in diabetes management. This study aimed at investigating the hypoglycemic and antidiabetic effects of PSC aqueous extract by evaluating the glucose tolerance test and certain important serum profiles in normal and STZ-induced diabetic rats.

## 2. Materials and Methods

### 2.1. Preparation of PSC Aqueous Extract

Fresh fruiting bodies of PSC (5 kg; pileus cap diameter between 9 and 11 cm) were obtained from National Kenaf and Tobacco Board (NKTB), Malaysia. It was identified and authenticated by the authority in NKTB, Malaysia. After drying process, Biodehydration (the secret trade drying process done by Anjaad industry, Malacca, Malaysia), the dehydrated PSC (yield: 10% w/w) were milled and sifted into fine powder having diameter of 125 *μ*m. For the aqueous extract preparation, 20 g of PSC powder was extracted with 250 mL of distilled water by using Soxhlet apparatus (hot continuous extraction method). This process was continuously carried out for 3 days until a drop of distilled water did not leave residue when evaporated. After the extract was cooled down, it was put into screw cap bottle and freeze-dried to obtain PSC aqueous extract (yield: 30% w/w).

### 2.2. Animals

Male Sprague-Dawley rats with body weight of 250–300 g each were supplied from Animal Research and Service Centre (ARASC), Universiti Sains Malaysia. They were housed in polypropylene cages (1 rat in each cage) in a room with controlled temperature (25°C ± 2°C) and maintained in a light : dark (12 : 12) cycle. The rats were provided with pellet diet and water* ad libitum*. Prior to experimentation, they were acclimatized for a period of one week. Male animals were chosen for most of the studies as the females have appeared to be secured from lipid-induced reductions in insulin action [16]. This study was permitted by the Universiti Sains Malaysia Animal Ethics Committee (USM/Animal Ethics Approval/2013/(85) (464)).

### 2.3. Induction of Diabetes

After one week of acclimatization, freshly prepared Streptozotocin (STZ) (40 mg/kg BW) in 0.1 M citrate buffer (pH 4.5) has been administrated to overnight-fasted rats by a single intraperitoneal injection to induce diabetes [17]. Fasting blood glucose (FBG) level was measured at the diabetes induction stage until stable hyperglycemia, usually one week after STZ administration. Diabetic rats were defined as those having FBG level exceeding 8 mmol/L.

### 2.4. Experimental Design

Initial screening of the PSC aqueous extract on examining its hypoglycemic potential was conducted by BG study. Variable doses of 500, 750, and 1000 mg/kg of extract were given orally by gavage in normal rats. Besides, oral glucose tolerance test (OGTT) was also performed in normal and diabetic rats with the mentioned doses of the extract. By identifying the most effective dose (750 mg/kg) of PSC aqueous extract from previous experiments, the antidiabetic effects of PSC aqueous extract were assessed for 21 days in diabetic rats. This experimental design was similar to that of studies described by [6, 18] with some modifications.

#### 2.4.1. Initial Screening of Hypoglycemic Activity of the Extract in Normal Rats

Thirty overnight-fasted normal rats were equally divided into five groups of six rats each. Group I (control) received vehicle (distilled water), whereas group II (positive control) received metformin (150 mg/kg). Variable doses of PSC aqueous extract (500, 750, and 1000 mg/kg) suspended in distilled water were administrated orally to groups III, IV, and V, respectively. Blood samples were then withdrawn at 2, 4, and 6 h to determine BG level after FBG measurement.

#### 2.4.2. Assessment of Hypoglycemic Activity of the Extract by OGTT in Normal Rats

Another group of thirty overnight-fasted normal rats were equally divided and treated on the previously mentioned approach. FBG was indicated at the beginning and BG level was measured after 90 min of treatment expressed as “0” h value. Oral administration of 0.2 g/mL glucose solution at 1 mL/100 g body weight (2 g/kg) was delivered to all of the groups. BG level was further measured, up to 3 h with 1 h intervals each, expressed as 1, 2, and 3 h values.

#### 2.4.3. Assessment of Hypoglycemic Activity of the Extract by OGTT in Diabetic Rats

The hypoglycemic potential of PSC aqueous extract in diabetic rats was evaluated in the glucose tolerance test. Thirty overnight-fasted diabetic rats were equally divided and also treated on the same pattern. The protocol in conducting the OGTT study in diabetic rats was the same as those for normal rats (in Section 2.4.2).

#### 2.4.4. Assessment of Antidiabetic Activity of the Extract in Diabetic Rats

Subacute study (21-day) of the effects of PSC aqueous extract on antidiabetic properties was conducted in diabetic rats. Twenty-four rats were equally divided into four groups of six rats each. Control groups (groups I and II) were normal and diabetic rats receiving vehicle (distilled water). Groups III and IV were both diabetic rats and treated with the effective dose of 750 mg/kg of the extract and 150 mg/kg of metformin as positive control, respectively. The allocated treatment was administered orally to all of the groups by gavage once daily in the morning for a period of 21 days. FBG, urine sugar, and body weight (BW) were measured at the beginning (0 days) and after 7, 14, and 21 days of the experiment. At the end of the experiment, five milliliters of blood was withdrawn by means of cardiac puncture (terminal bleeding) under general anaesthesia (pentobarbitone 100 mg/kg) from all of the experimental groups. Lipid profiles, renal function profiles, liver function profiles, and glycated haemoglobin (HbA1c) were also analysed after the experiment.

#### 2.4.5. LD_50_ Experiment

Acute toxicity of PSC aqueous extract was examined by conducting LD_50_ experiment. Sixteen normal rats were equally divided into two groups of eight rats each (4 males and 4 females). A single dose of either 5 (3.75 g/kg) or 10 (7.5 g/kg) times the PSC aqueous extract effective dose was orally administrated to them. The rats were then observed for gross behavioural, autonomic, neurologic, and toxic effect for 24 h with short intervals of time. Food consumption, faeces, and urine were also inspected at first 2 h and then at 6 h intervals for 24 h.

### 2.5. Estimations

Blood samples were withdrawn by means of tail artery prick method to measure BG level (mmol/L) using glucometer (Accu-Check Performa, Roche). Body weight (g) of rats was measured gravimetrically using analytical balance. Urine sugar levels were detected by Combur test from Roche Diagnostics. HbA1c (%) levels were determined by using Cobas Integra 800 analyser based on Turbidimetric Inhibition Immunoassay (TINIA) method. Lipid, renal function and kidney function profiles were determined by using Architect (Abbott brand, Ci8000) analyser based on enzymatic photometric method.

### 2.6. Statistical Analysis

Data obtained were tested for significance using two-way repeated measures analysis of variance (ANOVA) followed by Bonferroni's Multiple Comparison Test. The data were analysed by using GraphPad Prism version 6.0 for windows (GraphPad Software, San Diego, California, USA). Results were expressed as mean ± standard deviation. Significant differences have been only established if *p* < 0.05.

## 3. Results

### 3.1. Hypoglycemic Effect of PSC Aqueous Extract on BG in Normal Rats

Results from initial screening of hypoglycemic effect of graded doses of PSC aqueous extract in normal rats are presented in Table 1. Generally, all rats showed reduction on BG level 6 h after administration. The doses of 750 and 1000 mg/kg PSC extract produced significant (*p* < 0.05) reduction of BG level compared to vehicle control but established no significant difference compared to metformin as positive control. Hence, the PSC extract has been demonstrated to have similar hypoglycemic effect as that of metformin. Moreover, 6 h after administration, it was noticed that the rats treated with 750 mg/kg of PSC extract produced the highest maximum fall (23.5%) in BG level among all PSC extract-treated groups, whereas the fall of 11.8 and 21.2% was observed with the doses of 500 and 1000 mg/kg, respectively. In the aspect of time frame, 750 mg/kg dose started to report significant reduction in BG level 4 h after administration; meanwhile 1000 mg/kg dose showed significant reduction in BG level only 6 h after administration. It could be stated that dose of 750 mg/kg documented more immediate hypoglycemic effect than that of 1000 mg/kg. Hence, 750 mg/kg dose was selected to be the most effective dose of PSC extract from this BG study.

### 3.2. Hypoglycemic Effect of PSC Aqueous Extract on OGTT in Normal Rats

**Figure 1:**
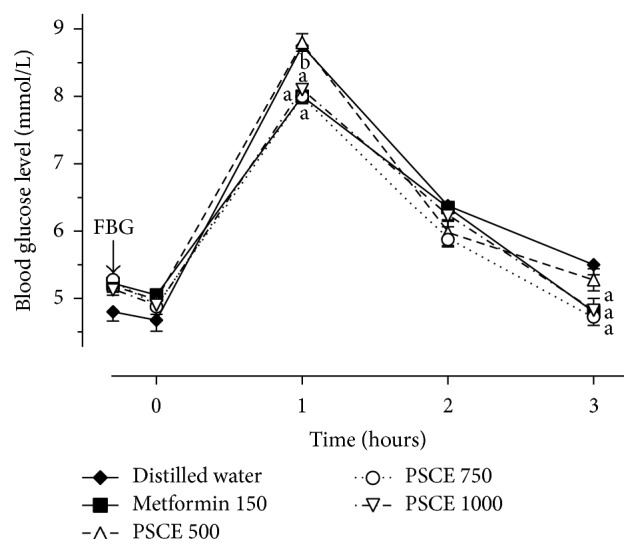
Hypoglycemic effect of graded doses of PSC aqueous extract on glucose tolerance in normal rats. ^a^ *p* < 0.05 as compared to normal control group (distilled water); ^b^ *p* < 0.05 as compared to normal treated group (metformin 150 mg/kg). PSCE,* Pleurotus sajor-caju* aqueous extract.

In order to determine the most effective dose of PSC aqueous extract on glucose tolerance in normal rats, variable doses (500, 750, and 1000 mg/kg) of PSC extract were examined along with metformin (150 mg/kg) as standard drug and the effects were depicted up to 3 h after glucose administration (Figure 1). Rats treated with the doses of 750 and 1000 mg/kg significantly (*p* < 0.05) produced the lower BG level peak in comparison to vehicle control group 1 hr after glucose loading. The effect of the doses also demonstrated no significant difference compared to metformin group. However, it was more apparent in rats receiving 750 mg/kg of the PSC extract. This dose brought a raise of 63.3%, which is the lowest increment of BG level compared to 76.0 and 65.3% in 500 and 1000 mg/kg, respectively. Apart from that, 3 h after glucose loading, BG level of rats treated with the doses of 750 and 1000 mg/kg PSC extract was reduced significantly (*p* < 0.05) compared to vehicle control but there was no significant difference compared to metformin. The dose of 750 mg/kg exhibited a maximum fall of 41.3%, whereas the fall of 39.8 and 40.7% was documented with the doses of 500 and 1000 mg/kg, respectively.

### 3.3. Hypoglycemic Effect of PSC Aqueous Extract on OGTT in Diabetic Rats

**Figure 2:**
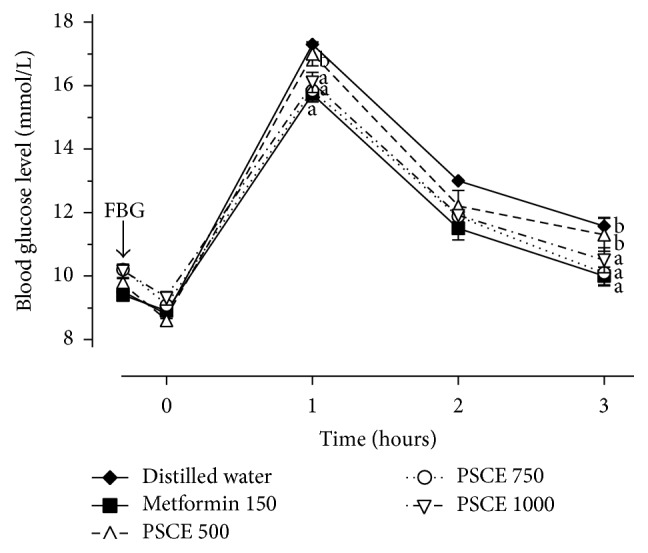
Hypoglycemic effect of graded doses of PSC aqueous extract on glucose tolerance in diabetic rats. ^a^ *p* < 0.05 as compared to diabetic control group (distilled water); ^b^ *p* < 0.05 as compared to diabetic treated group (metformin 150 mg/kg). PSCE,* Pleurotus sajor-caju* aqueous extract.

The above-mentioned variable doses of PSC extract were given to diabetic rats and the hypoglycemic effects were evaluated along with the standard drug metformin. The result of glucose tolerance test up to 3 h of glucose loading was illustrated in Figure 2. For the 500 mg/kg as lower dose, the BG level reported no significant difference compared to control treatment 1 h after glucose administration, indicating no hypoglycemic effect. However, diabetic rats treated with 750 and 1000 mg/kg of the PSC extract significantly (*p* < 0.05) exhibited the lower BG level peak compared to vehicle control group and produced no significant difference compared to metformin group. Besides, the BG level peak increment degree of 750 (74.7%) and 1000 mg/kg (73.1%) was also lower if compared to 500 mg/kg dose (97.7%). 3 h after glucose loading, BG level of rats treated with the doses of 750 and 1000 mg/kg PSC extract documented significant reduction (*p* < 0.05) compared to vehicle control but there was no significant difference compared to metformin. The dose of 750 mg/kg exhibited a maximum fall of 36.5%, whereas the fall of 33.5 and 34.8% was reported with the doses of 500 and 1000 mg/kg, respectively.

### 3.4. Antidiabetic Effects of PSC Aqueous Extract on FBG, Body Weight, Urine Sugar, and Several Important Serum Profiles in Diabetic Rats (Long Term Study)

It was attempted to indicate the effect of subacute repeated oral administration of the PSC extract on FBG, body weight, and urine sugar in STZ-induced diabetic rats (Table 2). The parameters were measured before and after 7, 14, and 21 days of treatment. Generally, the PSC extract and metformin exhibited gradual FBG reduction in diabetic rats, whereas FBG increment was detected in control diabetic group throughout the study. After 14-day treatment period, FBG of diabetic rats treated with PSC extract was decreased by 5% and further decreased by 6.9% after 21 days significantly (*p* < 0.05). The FBG reduction effect of metformin was shown better and earlier than the PSC extract, in which it started to show FBG reduction significantly after 7 days of treatment (7.7%; *p* < 0.05). In comparison to all groups, diabetic rats treated with PSC extract reported significant (*p* < 0.05) reduction of FBG compared to control diabetic rats after 14 and 21 days of treatment. At the same time, their FBG also showed significant difference (*p* < 0.05) compared to metformin group. It is indicated that the PSC extract possesses significant FBG reduction effect but the effect was not comparable to standard drug metformin.

In the aspect of body weight, metformin group showed body weight increment; meanwhile groups of control and PSC extract treatment reported gradual degradation of body weight throughout the study. Body weight of PSC extract-treated group showed significant (*p* < 0.05) degradation every 7 days, with 7.1% degradation after 21 days of treatment. In comparison to all groups, PSC extract-treated diabetic group reported no significant difference compared to control diabetic group after 7 and 14 days of extract treatment. Nevertheless, there was significant difference (*p* < 0.05) detected between control and PSC extract-treated groups after 21 days of treatment. The results indicated that 21-day treatment of PSC extract maintained body weight of rats which was dropped initially.

Basically, PSC extract-treated diabetic group showed lower urine sugar compared to control diabetic groups but still not comparable to metformin group during this subacute study. For PSC extract-treated diabetic group, the urine sugar was reduced by 50% after 14 days of extract treatment and maintained the fall until the end of experiment.

After blood was withdrawn by means of cardiac puncture (terminal bleeding) at the end of experiment, several serum profiles were determined and results of all groups were compared (Table 3). Lipid profiles (total cholesterol, HDL cholesterol, LDL cholesterol, and triglyceride) of diabetic rats treated with PSC extract reported no significant difference compared to control and metformin-treated diabetic rats. However, compared to diabetic control group, there was slight improvement shown on the lipid profiles even though no marked antihypercholesterolemic effects of PSC extract were observed in diabetic rats. Moreover, analysed renal function profiles (urea, creatinine, and uric acid) and liver function profiles (albumin, globulin, and total bilirubin) of all groups reported similar trend of result to that of lipid profiles, in which there was no significant difference compared to other groups. In terms of HbA1c, diabetic rats treated with PSC extract documented lower reading with 6.5% reduction observed compared to control diabetic rats. From the above experimental outcome, it could be stated that long term treatment of the PSC extract did not possess significant antidiabetic properties but it exhibited more or less improvement in terms of the important serum profiles in diabetes.

### 3.5. LD_50_ Experiment

Up to 5 and 10 times the effective dose of PSC aqueous extract, the behaviour of all the treated rats appeared to be normal. Furthermore, there was no toxic effect documented and no death occurred in all treated rats.

## 4. Discussion

The intention of this research was to investigate the effects of PSC aqueous extract on glycemic control, glucose tolerance, body weight, urine sugar, HbA1c, and lipid, renal function as well as liver function profiles. STZ-induced diabetic rat was used as a useful experimental model as it established diabetes properties [19]. Metformin (positive control) was used because it is one of the standard drugs used for management of diabetes.

*Pleurotus sajor-caju* aqueous extract reduced BG level in normal rats during initial screening. Besides, it also significantly ameliorated glucose tolerance in normal and diabetic rats by minimizing the high BG level peak after glucose loading. This indicated that PSC extract has favourable effects in bringing down the complications such as glucose intolerance and preventing severity of diabetes. From the above acute single administration study, the most optimum effective dose was 750 mg/kg which was selected for long term repeated treatment study. It is because 750 mg/kg dose showed the maximum reduction (23.5%) in BG level in FBG test and maximum improvement in OGTT particularly 1 and 2 h after administration of PSC extract. It is proposed thereby that the active compounds of the PSC extract or its metabolites may take approximately 120 minutes to perform their hypoglycemic effect in the target tissues. Moreover, it also produced comparable effect as of synthetic drug metformin (150 mg/kg). Nevertheless, the higher dose (1000 mg/kg) of PSC extract given did not report better hypoglycemic effects. The situation of less hypoglycemic potential at increasing dose is common with indigenous plant and has already been discovered in* Cynodon dactylon* [6],* Moringa oleifera* [18], and* Trichosanthes dioica* [20]. This phenomenon might be due to insufficient amount of active compound present in the extract [18].

During subacute treatment, significant improvement in FBG, body weight, and urine sugar was documented in diabetic rats treated with PSC extract. It was evidenced that FBG of diabetic rats treated with PSC extract was decreased significantly by 6.6% after 21 days and showed significant reduction compared to control diabetic rats within 14 days. Besides, body weight and urine sugar of PSC extract-treated group showed significant degradation (7.1%) after 21 days and decrement by 50% after 14 days of extract treatment, respectively. Elevated levels of FBG and urine sugar as well as reduced body weight are basically associated with diabetes. Hence, improvement of these diabetes characteristics indirectly justified the antidiabetic activity of the PSC extract.

Streptozotocin selectively damages pancreatic insulin-secreting *β*-cells leading to diabetes [19]. Occurrences of hyperlipidemia and kidney disease are the complications caused by diabetes [21]. For serum profiles analyses, lipid, renal function and liver function profiles of PSC extract-treated diabetic rats showed slight improvement compared to control diabetic rats despite being not significant. It is suggested that the PSC extract could bring more or less antidiabetic effects in the studied serum profiles. Hence, the PSC extract could be fundamental agent in overcoming complications caused by diabetes. This finding is in line with* Pleurotus ostreatus*, a type of oyster mushroom which has been examined to have beneficial effect on glycemic control and lipid profile [13]. Furthermore, a similar hypoglycemic effect was observed on* Agaricus bisporus* (white button mushroom). It has a positive influence on lipid metabolism and liver function [22]. In order to ensure that the PSC is safe to be consumed, acute toxicity study (LD_50_) was performed. High LD_50_ of PSC aqueous extract denotes its high margin of safety.

Nutritional investigation of PSC reveals the presence of high content of dietary fiber (35.6%) including *β*-glucan (3.57%) [23]. These principles are recognized to be hypoglycemic and antidiabetic agents in diabetes management. Dietary fiber and *β*-glucan have scientifically proven its synergistic effects and protective effects against various diseases such as heart disease, stroke, and diabetes as they have the potential to play an adjunctive role in reducing cholesterol and BG levels [24]. In detail, *β*-glucan is a soluble dietary fiber which can form viscous solution; thus it is resistant to stomach digestive enzyme and delays glucose absorption in intestine as well as gastric emptying rate [25]. Therefore, it may slow down the rise in BG level after administration of PSC aqueous extract, resulting in improvement of glucose tolerance. Apart from that, glucan-rich polysaccharides from PSC were also reported to prevent glucose intolerance, insulin resistance, and inflammation in mice fed with high-fat diet [26].

## 5. Conclusion

The present study concludes that PSC aqueous extract has demonstrated significant effects on BG level and apparent improvement on glucose tolerance in single administration study. This means that the PSC extract could give immediate hypoglycemic effect such as reduced postprandial glycemic response. For the subacute study with daily repeated administration of the extract, there were also some improvements or less severe conditions in all measured parameters. In addition, the PSC extract has been proven to be safely consumed even up to 10 times the most effective dose. These results evidently express the possible benefits of PSC aqueous extract in controlling diabetes and preventing its complications. Further biochemical, pharmacological, and clinical investigations should be undertaken to elucidate the possible mechanism of the hypoglycemic and antidiabetic activities of PSC aqueous extract.

## Figures and Tables

**Table 1:** Initial screening (BG study) of hypoglycemic effect of graded doses of PSC aqueous extract in normal rats (mean ± SD).

Experimental animals	Treatment	Blood glucose levels (mmol/L)			
		Pretreatment level	Posttreatment level		
		FBG	2 h	4 h	6 h
Normal (control)	Distilled water	5.0 ± 0.1	4.9 ± 0.1	4.8 ± 0.1	4.7 ± 0.1
Normal (treated)	Metformin (150 mg/kg)	5.3 ± 0.3	4.6 ± 0.1	4.2 ± 0.1^a^	4.0 ± 0.1^a^
Normal (treated)	PSCE (500 mg/kg)	5.1 ± 0.2	4.8 ± 0.1	4.6 ± 0.2^b^	4.5 ± 0.1^b^
Normal (treated)	PSCE (750 mg/kg)	5.1 ± 0.2	4.4 ± 0.2	4.1 ± 0.3^a^	3.9 ± 0.2^a^
Normal (treated)	PSCE (1000 mg/kg)	5.2 ± 0.3	4.6 ± 0.2	4.3 ± 0.1	4.1 ± 0.1^a^

^a^ *p* < 0.05 as compared to normal control group (distilled water).

^b^ *p* < 0.05 as compared to normal treated group (metformin 150 mg/kg).

PSCE, *Pleurotus sajor-caju* aqueous extract.

**Table 2:** Antidiabetic effects of oral administration of PSC aqueous extract on FBG, body weight, and urine sugar in diabetic rats (mean ± SD).

Experimental animals	Treatment	Pretreatment level	Posttreatment level		
		0 days	7 days	14 days	21 days
FBG (mmol/L)					
Normal (control)	Distilled water	5.5 ± 0.2	5.7 ± 0.2	5.4 ± 0.1	5.5 ± 0.2
Diabetic (control)	Distilled water	10.2 ± 0.3	10.4 ± 0.2	10.2 ± 0.3	10.9 ± 0.4
Diabetic (treated)	Metformin (150 mg/kg)	10.4 ± 0.2	^p^9.6 ± 0.1^a^	^pq^8.9 ± 0.3^a^	^p^8.9 ± 0.3^a^
Diabetic (treated)	PSCE (750 mg/kg)	10.1 ± 0.2	10.0 ± 0.3	^p^9.6 ± 0.2^ab^	^pq^9.4 ± 0.2^ab^
Body weight (g)					
Normal (control)	Distilled water	299.3 ± 9.9	327.3 ± 9.5	^pq^364.8 ± 4.1	^pqr^403.3 ± 6.7
Diabetic (control)	Distilled water	279.3 ± 5.4	270.5 ± 3.1	^p^258.3 ± 7.4	^pqr^245.5 ± 6.0
Diabetic (treated)	Metformin (150 mg/kg)	282.3 ± 2.5	^p^295.8 ± 2.9^a^	^p^308.3 ± 2.2^a^	^pqr^318.5 ± 1.3^a^
Diabetic (treated)	PSCE (750 mg/kg)	275.8 ± 4.6	^p^269.5 ± 4.7^b^	^pq^264.5 ± 4.7^b^	^pqr^256.3 ± 2.1^ab^
Urine sugar (g/L)					
Normal (control)	Distilled water	0	0	0	0
Diabetic (control)	Distilled water	+2	+2	+2	+2
Diabetic (treated)	Metformin (150 mg/kg)	+2	+1	0	0
Diabetic (treated)	PSCE (750 mg/kg)	+2	+2	+1	+1

^a^ *p* < 0.05 as compared to diabetic control group (distilled water).

^b^ *p* < 0.05 as compared to diabetic treated group (metformin 150 mg/kg).

^p^ *p* < 0.05 as compared to pretreatment level (0 days).

^q^ *p* < 0.05 as compared to 7 days.

^r^ *p* < 0.05 as compared to 14 days.

PSCE, *Pleurotus sajor-caju* aqueous extract.

**Table 3:** Antidiabetic effects of oral administration of PSC aqueous extract on lipid profiles, liver function profiles, kidney function profiles, and HbA1c in mild diabetic rats (mean ± SD).

Experimental animals	Treatment	21 days Posttreatment level
Total cholesterol (mmol/L)		
Normal (control)	Distilled water	1.5 ± 0.0
Diabetic (control)	Distilled water	1.9 ± 0.2
Diabetic (treated)	Metformin (150 mg/kg)	1.7 ± 0.1
Diabetic (treated)	PSCE (750 mg/kg)	1.8 ± 0.1
HDL cholesterol (mmol/L)		
Normal (control)	Distilled water	1.2 ± 0.1
Diabetic (control)	Distilled water	1.5 ± 0.1
Diabetic (treated)	Metformin (150 mg/kg)	1.4 ± 0.1
Diabetic (treated)	PSCE (750 mg/kg)	1.4 ± 0.2
LDL cholesterol (mmol/L)		
Normal (control)	Distilled water	0.2 ± 0.1
Diabetic (control)	Distilled water	0.4 ± 0.1
Diabetic (treated)	Metformin (150 mg/kg)	0.2 ± 0.1
Diabetic (treated)	PSCE (750 mg/kg)	0.3 ± 0.0
Triglyceride (mmol/L)		
Normal (control)	Distilled water	0.5 ± 0.2
Diabetic (control)	Distilled water	0.6 ± 0.2
Diabetic (treated)	Metformin (150 mg/kg)	0.5 ± 0.1
Diabetic (treated)	PSCE (750 mg/kg)	0.6 ± 0.1
Urea (mmol/L)		
Normal (control)	Distilled water	7.0 ± 0.5
Diabetic (control)	Distilled water	8.1 ± 0.3
Diabetic (treated)	Metformin (150 mg/kg)	7.4 ± 0.5
Diabetic (treated)	PSCE (750 mg/kg)	8.1 ± 0.3
Creatinine (*μ*mol/L)		
Normal (control)	Distilled water	51.8 ± 2.6
Diabetic (control)	Distilled water	61.6 ± 0.0
Diabetic (treated)	Metformin (150 mg/kg)	54.6 ± 2.3
Diabetic (treated)	PSCE (750 mg/kg)	58.3 ± 2.1
Uric acid (*μ*mol/L)		
Normal (control)	Distilled water	188.3 ± 13.1
Diabetic (control)	Distilled water	192.2 ± 13.0
Diabetic (treated)	Metformin (150 mg/kg)	191.8 ± 10.4
Diabetic (treated)	PSCE (750 mg/kg)	190.7 ± 15.6
Albumin (g/L)		
Normal (control)	Distilled water	25.1 ± 1.6
Diabetic (control)	Distilled water	22.4 ± 1.5
Diabetic (treated)	Metformin (150 mg/kg)	23.7 ± 1.6
Diabetic (treated)	PSCE (750 mg/kg)	22.1 ± 1.8
Globulin (g/L)		
Normal (control)	Distilled water	38.7 ± 1.9
Diabetic (control)	Distilled water	34.0 ± 2.3
Diabetic (treated)	Metformin (150 mg/kg)	35.9 ± 2.5^a^
Diabetic (treated)	PSCE (750 mg/kg)	35.1 ± 2.6
Total bilirubin (*μ*mol/L)		
Normal (control)	Distilled water	1.7 ± 0.0
Diabetic (control)	Distilled water	3.1 ± 0.2
Diabetic (treated)	Metformin (150 mg/kg)	1.7 ± 0.0^a^
Diabetic (treated)	PSCE (750 mg/kg)	2.7 ± 0.5
HbA1c (%)		
Normal (control)	Distilled water	3.7 ± 0.1
Diabetic (control)	Distilled water	6.2 ± 0.3
Diabetic (treated)	Metformin (150 mg/kg)	3.9 ± 0.2^a^
Diabetic (treated)	PSCE (750 mg/kg)	5.8 ± 0.5

^a^ *p* < 0.05 as compared to diabetic control group (distilled water).

PSCE, *Pleurotus sajor-caju* aqueous extract.
